# Supplementary material for: Calprotectin as a sepsis diagnostic marker in critical care: a retrospective observational study
Source: Sci Rep. 2025 May 3;15:15529. doi: 10.1038/s41598-025-95420-0 (PMC12049440; doi:10.1038/s41598-025-95420-0)
Supplement: Supplementary file 1 — Supplementary Information 1. [file 41598_2025_95420_MOESM1_ESM.pdf]

Supplement 1: Calprotectin as a sepsis diagnostic marker in critical care: a retrospective observational study

Contents

1 Methods supplement **1**
1.1 Definition of clinically relevant pathogens in microbiological samples 1
1.2 Classification of pathogens 2
1.3 Stratification of patients with culture-positive sepsis into one of five subgroups . 2
1.4 All pathogens found in microbiological cultures/tests 4

2 Results supplement **12**
2.1 Droput analysis 12
2.2 Sample handling times 13
2.3 Calprotectin levels in subgroups of ICU patients. 13
2.4 Calprotectin and CRP levels in microbiological subgroups of sepsis patients. . . 13
2.5 Association between calprotectin and CRP in microbiological subgroups of sepsis patients. 14

1 Methods supplement

1.1 Definition of clinically relevant pathogens in microbiological samples

Microbiological cultures were defined as clinically relevant if there was growth or detection of a pathogen from a culture taken within the stipulated time frame, regardless of the anatomical site of culturing. The following culture results were considered clinically irrelevant:

1. Yeast fungi from non-sterile anatomical sites (e.g. airways, lower urinary tract, skin lesions)

2. Potentially colonising bacteria of the upper or lower respiratory tract, if found in only one airway culture: Moraxella sp., coagulase-negative Staphylococci, viridans (alpha hemolytic) Streptococci

3. Potential skin contaminants found in only one blood culture: coagulase-negative Staphylococci, viridans (alpha hemolytic) Streptococci, micrococcus sp., Propionibacterium Acnes, Corynebacterium sp., Bacillus sp. [1]

4. Unspecific culture results (e.g. "gram-positive mixed flora", "vaginal flora", "skin flora", "anaerobic mixed flora") from non-sterile anatomical sites

5. Bacterial growth of Clostridium difficile, without detection of toxin[2]

6. Pneumococcus antigen tests from urinary samples

1.2 Classification of pathogens

Pathogens were categorized into gram-positive bacteria, gram-negative bacteria, viruses, and fungi. Bacteria were further categorised by family or species. Atypical bacteria were considered gram-negative bacteria in this study. Viruses and fungi were categorized as seen below. *Sp; species.*

Gram-negative bacteria

- Acinetobacter sp.
- Bacteroides sp.
- Escherica coli
- Fusobacterium sp.
- Haemophilus influenzae
- Klebsiella sp.
- Moraxella sp.
- Neisseria meningitidis
- Other Enterobacter sp.
- Pseudomonas aeruginosa
- Stenotrophomonas maltophilia
- Other gram-negative bacteria
- Unspecified gram-negative bacteria
- Atypical bacteria

Fungi

- Candida sp.
- Pneumocystis sp.
- Other fungi

Viruses

- Influenza type A and B
- Other viruses

Gram-positive bacteria

- Bacillus sp.
- Clostridium difficile
- Coagulase-negative staphylococci
- Corynebacterium sp.
- Cutibacterium acnes
- Enterococcus faecalis
- Enterococcus faecium
- Lactobacillus sp.
- Mycobacterium sp.
- Staphylococcus aureus
- Streptococcus pneumoniae
- Streptococcus pyogenes
- Viridans streptococci
- Other Clostridium
- Other Enterococci
- Other Betastreptococci
- Other Streptococci
- Other gram-positive bacteria
- Unspecified gram-positive bacteria

1.3 Stratification of patients with culture-positive sepsis into one of five subgroups

Microbiological cultures and samples were filtered by if they were taken within ± 48 of ICU admission and if they were considered clinically relevant.

Based on the culture results, all sepsis patients were sorted into one of five categories. The five categories were gram-positive bacteria, gram-negative bacteria, fungal, viral and polymicrobial. The polymicrobial patient could have the following combinations of pathogens:

- Mixed bacteria (meaning cultures contain both gram-positive bacteria and gram-negative bacteria)
- Gram-negative bacteria + fungi or virus
- Gram-positive bacteria + fungi or virus
- Mixed bacteria + fungi or virus
- Fungi + virus

Most of the patients had cultures containing only one group of pathogens (i.e. gram-positive bacteria, gram-negative bacteria, fungi or viruses). However, about a third of the patients had cultures belonging to more than one of these groups. These were sorted as follows:

1. If the patient had a positive blood culture, the type of pathogen growing in the blood culture decided which group the patient belonged to. If more than one type of pathogen was found in blood cultures, the patient was allocated to the polymicrobial cohort. If the patient, in addition to positive blood cultures, tested positive for viruses (with any method), they were also categorised as polymicrobial. A few exceptions were made when there was a significantly higher occurrence of one type of pathogen type among the cultures from one patient.
2. If there were no blood cultures, the type of pathogen and the anatomic location it was sampled from were considered next. With the help of Infectious Diseases specialist LM (co-author), the following hierarchy was decided upon:
   (a) If primary pathogens (see below) were found in their respective anatomic location, the type of that pathogen decided which group the patient belonged to. If more than one type of primary pathogen was found, the patient was assigned to the polymicrobial group.
   (b) Other significant pathogens were considered if no primary pathogens were found.
   (c) Viruses were always considered relevant.
   (d) Fungi (other than candida sp. in upper respiratory specimen) were considered relevant when found alone in the lungs or the abdomen without other relevant pathogens.

Primary pathogens:

- Urinary tract: Escherica coli, Proteus sp, Klebsiella and other enterobacter sp.
- Respiratory tract: Streptococcus pneumoniae, Haemophilus influenzae, Legionella sp. Mycoplasma pneumoniae.
- All cultures from the central nervous system, bones, and joints
- Group A streptococcus and meningococcus sp: always significant regardless of anatomical site.

Significant pathogens:

- Enterococcus faecium in abdomen.
- Enterococcus faecalis in abdomen. When found in urine without other significant pathogens elsewhere.

- Pseudomonas sp. in airways or urine
- Staphylococcus Aureus in any other location besides the urinary tract unless it coexists with the primary pathogen in the respiratory tract.
- Group C and G streptococcus in airways, skin, and soft tissue.

Non-significant pathogens (unless no other significant pathogens were found)

- Enterococcus faecium in urine. Enterococcus faecium and faecalis in the respiratory tract.
- Moraxella, Coagulase negative staphylococcus, corynebacteria, acinetobacter, propionibacterium, stenotrophomonas sp.
- Staphylococcus Aureus in urine
- Group B streptococcus
- Viridans streptococcus

1.4 All pathogens found in microbiological cultures/tests

S 1: All pathogens found in cultures and other microbiological tests sorted by gram stain (bacteria) and family or species (all pathogens). In some cases, the laboratory was unable to identify bacteria further than by general characteristics.

Family or species	Pathogen
Gram-negative bacteria	
**Acinetobacter sp.**	Acinetobacter baumannii
	Acinetobacter baumannii-calcoaceticus complex
	Acinetobacter pittii
	Acinetobacter sp.
	Acinetobacter ursingii
**Bacteroides sp.**	Bacteroides caccae
	Bacteroides fragilis
	Bacteroides ovatus
	Bacteroides sp.
	Bacteroides thetaiota omicron
	Bacteroides uniformis
	Bacteroides vulgatus
**Escherichia Coli**	Escherichia coli
**Fusobacterium sp.**	Fusobacterium gonidia formans
	Fusobacterium necrophorum
	Fusobacterium nucleatum
	Fusobacterium sp.
**Haemophilus**	Haemophilus influenzae
	Haemophilus parainfluenzae
	Haemophilus sp.
**Klebsiella sp.**	Klebsiella-Enterobacter sp.
	Klebsiella aerogenes
	Klebsiella oxytoca

	Klebsiella oxytoca group
	Klebsiella pneumoniae
	Klebsiella sp.
**Moraxella sp.**	Moraxella catarrhalis
	Moraxellanon liquefaciens
**Neisseria meningitidis**	Neisseria meningitidis
**Other enterobacter**	Citrobacter freundii
	Citrobacter koseri
	Citrobacter sp.
	Enterobacter asburiae
	Enterobacter cloacae
	Enterobacter cloacae complex
	Enterobacter sp.
	Enterobacterales
	Hafnia alvei
	Morganella morganii
	Pantoea agglomerans
	Pantoea sp.
	Proteus mirabilis
	Proteus penneri
	Proteus sp.
	Proteus vulgaris
	Proteus vulgaris group
	Providencia rettgeri
	Salmonella dublin
	Salmonella enteritidis
	Salmonella florida
	Salmonella group O10
	Salmonella sp.
	Salmonella subsp.
	Salmonella weltevreden
	Serratia liquefaciens
	Serratia marcescens
	Serratia rubidaea
	Serratia sp.
**Pseudomonas aeruginosa**	Pseudomonas aeruginosa
**Stenotrophomonas maltophilia**	Stenotrophomonas maltophilia
**Other gram-negative bacteria**	Achromobacter sp.
	Achromobacter xylosoxidans
	Aeromonas hydrophila
	Aeromonas sp.
	Alcaligenes faecalis
	Alloscardovia omnicolens
	Bordetella bronchiseptica
	Burkholderia cenocepacia
	Burkholderia cepacia
	Burkholderia cepacia complex
	Burkholderia gladioli

	Campylobacter jejuni/coli
	Campylobacter ureolyticus
	Capnocytophaga sp.
	Chryseobacterium gleum
	Chryseobacterium indologenes
	Chryseobacterium sp.
	Comamonas testosteroni
	Cronobacter sakazakii
	Cyberlindner afabianii
	Delftia acidovorans
	Dermabacter hominis
	Dialister pneumosintes
	Eikenella corrodens
	Elizabethkingia meningoseptica
	Elizabethkingia miricola
	Elizabethkingia sp.
	Hungatella hathewayi
	Leclercia adecarboxylata
	Lichtheimia corymbifera
	Neisseria bacilliformis
	Neisseria sp.
	Neisseria subflava
	Ochrobactrum anthropi
	Ochrobactrum sp.
	Pasteurella multocida
	Porphyromonas endodontalis
	Porphyromonas somerae
	Porphyromonas sp.
	Prevotella bivia
	Prevotella denticola
	Prevotella melaninogenica
	Prevotella nigrescens
	Prevotella oralis
	Prevotella oris
	Prevotella sp.
	Pseudomonas sp.
	Sphingomonas paucimobilis
	Sphingomonas sp.
	Sutterella wadsworthensis
	Veillonella parvula
	Veillonella sp.
	Vibrio sp.
	Vibrio vulnificus
**Atypical bacteria**	Legionella longbeachae
	Legionella pneumophila
	Legionella sp.
	Mycoplasma hominis
	Chlamydophila psittaci (DNA)

**Unspecified gram-negative bacteria**	Anaerobe gram-negative rods
	Gram-negative mixed flora
	Gram-negative mixed flora, e.g proteus
	Gram-negative mixed flora, e.g pseudomonas
	Gram-negative mixed environmental bacteria
Gram-positive bacteria	
**Bacillus sp.**	Bacillus cereus
	Bacillus cereus complex
	Bacillus sp.
**Clostridium Difficile**	Clostridium difficile toxin Vidas
	Clostridium difficile bacteria
**Coagulase-negative staphylococci**	Staphylococcus capitis
	Staphylococcus epidermidis
	Staphylococcus haemolyticus
	Staphylococcus hominis
	Staphylococcus lugdunensis
	Staphylococcus pasteuri
	Staphylococcus pettenkoferi
	Staphylococcus pseudintermedius
	Staphylococcus saprophyticus
	Staphylococcus schleiferi
	Staphylococcus simulans
	Staphylococcus sp. (coagulase-negative)
**Corynebacterium sp.**	Corynebacterium pseudodiphtheriticum
	Corynebacterium sp.
	Corynebacterium striatum
	Corynebacterium tuberculostearicum
**Cutibacterium acnes**	Cutibacterium acnes
**Enterococcus faecalis**	Enterococcus faecalis
**Enterococcus faecium**	Enterococcus faecium
**Lactobacillus sp.**	Lactobacillus gasseri
	Lactobacillus plantarum
	Lactobacillus rhamnosus
	Lactobacillus sp.
	Lactococcus sp.
**Mycobacteria**	Mycobacteria seen in microscope
	Mycobacterium tuberculosis
**Staphylococcus aureus**	Staphylococcus aureus
**Streptococcus pneumoniae**	Streptococcus pneumoniae
	Likely pneumococci
**Streptococcus pyogenes**	Streptococcus pyogenes(GAS)
**Viridans streptococci**	Streptococcus anginosus
	Streptococcus anginosus complex
	Streptococcus massiliensis
	Streptococcus mitis
	Streptococcus mitis complex
	Streptococcus mutans complex
	Streptococcus oralis

	Streptococcus parasanguinis
	Streptococcus salivarius complex
	Streptococcus sanguinis complex
	Streptococcus vestibularis
**Other Clostridium**	Clostridium aldenense
	Clostridium butyricum
	Clostridium cadaveris
	Clostridium innocuum
	Clostridium paraputrificum
	Clostridium perfringens
	Clostridium ramosum
	Clostridium septicum
	Clostridium sp.
	Clostridium symbiosum
	Clostridium tertium
**Other enterococci**	Enterococcus avium
	Enterococcus casseliflavus
	Enterococcus durans
	Enterococcus gallinarum
	Enterococcus gallinarum group
	Enterococcus hirae
	Enterococcus raffinosus
	Enterococcus sp.
**Other beta streptococci**	Beta streptococci group C
	Beta streptococci group G
	Streptococcus agalactiae (group B)
	Streptococcus dysgalactiae
	Streptococcus dysgalactiae (group C)
	Streptococcus dysgalactiae (group G)
**Other streptococci**	Streptococcus bovis
	Streptococcus bovis complex
	Streptococcus sp.
**Other gram-positive bacteria**	Actinomyces odontolyticus
	Actinomyces sp.
	Actinomyces turicensis
	Actinotignum sanguinis
	Actinotignum schaalii
	Actinotignum sp.
	Aerococcus sanguinicola
	Aerococcus sp.
	Aerococcus urinae
	Aggregatibactera phrophilus
	Aggregatibacter sp.
	Alistipesonder donki
	Anaerococcus sp.
	Aureobasidium pullulans
	Bifidobacterium breve
	Bifidobacterium sp.

	Brevundimonas aurantiaca
	Brevundimonas sp.
	Cutibacterium avidum
	Cutibacterium granulosum
	Cutibacterium sp.
	Desulfovibrio desulfuricans
	Dialister sp.
	Difteroida stavar
	Eggerthella lenta
	Eggerthella sp.
	Eggerthiaca tenaformis
	Enterocloster clostridioformis
	Eubacterium sp.
	Facklamia hominis
	Finegoldi amagna
	Galactomyces candidus
	Gemella haemolysans
	Gemella morbillorum
	Gemella sp.
	Globicatella sp.
	Grampositiva kocker
	Granulicatella adiacens
	Granulicatella sp.
	Listeria innocua
	Listeria monocytogenes
	Massilia sp.
	Micrococcus luteus
	Micrococcus sp.
	Nocardia sp.
	Odoribacter splanchnicus
	Paenibacillus sp.
	Paeniclostridium sordellii
	Parabacteroides distasonis
	Parabacteroides sp.
	Paracoccus sp.
	Parvimonas micra
	Peptoniphilus harei
	Peptoniphilus sp.
	Peptostreptococcus anaerobius
	Peptostreptococcuss tomatis
	Pichia cactophila
	Pichia norvegensis
	Rothia mucilaginosa
	Rothia sp.
	Ruminococcus gnavus
	Solobacterium moorei
	Staphylococcus warneri
	Weissella sp.

**Unspecified gram-positive bacteria**	Anaerobe gram-positive rods
	Gram-positive cocci, likely streptococci or enterococci
	Gram-positive rods
	Gram-positive mixed flora
Unspecified gram stain	
**Unspecified**	Anaerobe bacteria
	Anaerobe mixed flora
	Bacteria
	Mixed flora
	Mixed flora, e.g proteus
	Mixed flora, e.g pseudomonas
	Fecal flora
	Skin flora
	Oral flora
	Oropharyngeal flora
	Vaginal flora
Fungi	
**Candida**	Candida albicans
	Candida dubliniensis
	Candida glabrata
	Candida inconspicua
	Candida krusei
	Candida parapsilosis
	Candida sp.
	Candida tropicalis
	Candida utilitis
**Pneumocystis**	Pneumocystis
**Other fungi**	Aspergillus fumigatus
	Aspergillus nidulans
	Aspergillus sp.
	Aspergillus terreus
	Clavispora lusitaniae
	Diutinaca tenulata
	Diutinaca tenulata
	Fusarium solani
	Fusarium sp.
	Geotrichum sp.
	Yeast fungi
	Yeast fungi, not C. albicans
	Kluyveromyces marxianus
	Magnusiomyces capitatus
	Malassezia sp.
	Meyerozyma guilliermondii
	Mold fungi
	Mucor circinelloides
	Rhizopus microsporus
	Saccharomyces cerevisiae
	Thread fungus

	Trichosporona sahii
	Wickerhamomyces anomalus
Viruses	
**Influenza**	Influenzae A
	Influenzae B
**Other viruses**	Varicella zoster virus
	RS-virus type B
	RS-virus type A
	Rhinovirus
	Parainfluenzae
	Norovirus I
	Norovirus II
	Metapneumovirus
	Herpes simplex virus 1
	Enterovirus
	Coronavirus
	Cytomegalovirus
	Adenovirus

2 Results supplement

2.1 Dropout analysis

Table of dropout analysis of included and excluded patients. The excluded patients did not have blood samples in the biobank. CRP in this table is from routine laboratory analysis. All variables, except outcomes, are at ICU admission. CRRT and mechanical ventilarion are during the ICU stay. *ICU; intensive care unit. LOS; length of stay. ER; emergency room. IQR; interquartile range. SD; standard deviation. CRP; C-reactive protein. WBC; white blood cell count. SAPS-3; simplified acute physiology score-3. SOFA; sequential organ failure assessment. CRRT; continuous renal replacement therapy.*

	Included	Excluded	p-value
n	4732	804	
Age, years (median [IQR])	68 [56-75]	66 [50-74]	<0.001
Male sex (%)	2856 (60)	467 (58)	0.24
ICU admission >48h from hospital admission (%)	1196 (25)	264 (33)	<0.001
Admitted from ER (%)	1956 (41)	294 (37)	0.012
Biomarkers			
C-reactive protein, mg/L (median [IQR])	43 [7-144]	42 [7-128]	0.28
Lactate, mmol/L (median [IQR])	2.0 [1.2-4.1]	1.8 [1-4.4]	0.031
WBC, 10^9/L (median [IQR])	12.9 [8.7-18]	12.6 [9.1-18.7]	0.4
Physiological parameters			
Sepsis (%)	2071 (44)	260 (32)	<0.001
Shock (%)	1520 (32)	207 (27)	0.004
SAPS-3 and SOFA score at ICU admission			
SAPS-3 score (median [IQR])	64 [52-75]	62 [49-74]	0.008
SOFA score (mean (SD))	7.2 (4)	6.4 (4.2)	<0.001
Outcomes			
ICU LOS (median [IQR])	2.5 [1.5-4.8]	2.2 [1.2-4.4]	<0.001
ICU mortality (%)	871 (18.4)	175 (21.8)	0.028
30-day mortality (%)	1435 (30)	252 (35)	0.008
CRRT (%)	627 (13)	87 (11)	0.065
Invasive mechanical ventilation (%)	3029 (64)	479 (60)	0.018

2.2 Sample handling times

The median time from ICU admission to freezing of the biobank sample was 1.6 hours (IQR 1.3-2.1 hours). Among the samples with missing data at the time of freezing (n=45), the median time from ICU admission to the refrigerator was 2.2 hours (IQR 1.6-3.0).

2.3 Calprotectin levels in subgroups of ICU patients.

Calprotectin levels are displayed as median (IQR) for the non-sepsis and sepsis groups within the subgroups of ICU patients. Community or hospital-acquired disease was defined as a hospital LOS under respective over 48 hours before ICU admission. Shock was defined as vasopressor need and a lactate >2 mmol/L. Suspected infection was defined as an obtained blood culture and administered antibiotics around the time of ICU admission. *ICU; intensive care unit. LOS; length of stay. n; number. IQR; interquartile range.*

	n	Sepsis (%)	Non-sepsis median (IQR)	Sepsis median (IQR)	p-value
Full ICU cohort	*4732*	*44%*	*1.3 (0.6-2.8)*	*2.1 (1.0-4.3)*	<0.001
Community-acquired	3536	43%	1.1 (0.5-2.4)	1.9 (0.9-3.9)	<0.001
Hospital-acquired	1196	45%	2.1 (1.0-4.5)	2.6 (1.2-5.6)	0.003
Neutropenic	83	82%	0.6 (0.3-3)	1.0 (0.2-3.5)	0.91
Age<65 years	1899	46%	1.3 (0.6-2.6)	2.1 (1-4.1)	<0.001
Age≥65 years	2833	41%	1.4 (0.5-3.1)	2 (0.9-4.6)	<0.001
Shock	1520	51%	1.6 (0.8-3.1)	2.3 (1.1-4.4)	<0.001
SOFA≥2	4329	49%	1.4 (0.6-2.9)	2.1 (1-4.3)	<0.001
Suspected infection	2008	77%	1.4 (0.6-3.2)	2.2 (1-4.7)	<0.001

2.4 Calprotectin and CRP levels in microbiological subgroups of sepsis patients.

The microbiological subgroups of sepsis patients were based on relevant culture results in ±48 hours from ICU admission. Patients were assigned to only one of the six microbiological sepsis subgroups (gram-positive, gram-negative, fungal, virus, polymicrobial and culture-negative). All microbiological sepsis subgroups had higher calprotectin and CRP levels than the non-sepsis group ($p<0.05$). When the sepsis group was divided into bacteriemia and non-bacteriemia, the difference in calprotectin and CRP levels was significant with a p-value<0.001. *n; number. IQR; interquartile range.*

Microbiological subgroup	n	Calprotectin, median (IQR)	CRP, median (IQR)
Non-sepsis	*2661*	*1.3 (0.6-2.8)*	17 (3-69)
Gram-positive	613	1.9 (1.0-4.5)	88 (18-188)
Gram-negative	697	1.9 (0.8-3.7)	81 (19-174)
Fungal	44	3.0 (1-5.6)	108 (40-218)
Virus	92	2.3 (1.1-4.5)	69 (31-153)
Polymicrobial	236	2.8 (1.3-6.2)	119 (39-205)
Culture-negative	389	2.1 (1-4.3)	118 (51-196)
Bacteriemic	539	2.6 (1.2-5.7)	143 (74-240)
Non-bacteriemic	1532	1.9 (0.9-3.8)	75 (18-164)

2.5 Association between calprotectin and CRP in microbiological subgroups of sepsis patients.

The Spearman's r is displayed for the non-sepsis patients and for each microbiological sugroup of sepsis patients. All correlations were statistically significant with $p<0.05$. The number (n) of patients in each subgroup is the same as in table 2.4 above. *CRP; C-reactive protein.*

Microbiological subgroup	Spearman's r
Full ICU cohort	0.47
Non-sepsis	0.41
Gram-positive	0.51
Gram-negative	0.40
Fungal	0.41
Virus	0.57
Polymicrobial	0.54
Culture-negative	0.41

References

[1] Alcorn K, Meier F, Schifman R. Blood Culture Contamination: Q-tracks 2013. College of American Pathologists. 2013.

[2] Crobach M, Planche T, Eckert C, Barbut F, Terveer E, Dekkers O, et al. European Society of Clinical Microbiology and Infectious Diseases: update of the diagnostic guidance document for Clostridium difficile infection. Clinical microbiology and infection. 2016;22:S63-81.
